# Global genomic landscape of *Staphylococcus lugdunensis*: population structure, antimicrobial resistance, and virulence determinants

**DOI:** 10.1128/aem.01893-25

**Published:** 2026-02-23

**Authors:** Donghong Yang, Pengcheng Du, Fang Wang, Shining Fu, Wentao Ni, Wen Xi, Ying Zhang, Chunyu Liu, Zhancheng Gao, Yukun He, Ran Li

**Affiliations:** 1 Department of Respiratory and Critical Care Medicine, Peking University People’s Hospital 572420, Beijing, China; 2 Medical Research Center, Beijing Institute of Respiratory Medicine and Beijing Chao-Yang Hospital, Capital Medical University 74639, Beijing, China; 3 Department of Respiratory and Critical Care Medicine, Beijing Jishuitan Hospital, Capital Medical University 66526, Beijing, China; Centers for Disease Control and Prevention, Atlanta, Georgia, USA

**Keywords:** *Staphylococcus lugdunensis*, whole genome sequencing, molecular typing, resistance gene, virulence gene

## Abstract

*Staphylococcus lugdunensis*, a coagulase-negative staphylococcus, has emerged as an opportunistic pathogen causing severe infections. It exhibits genetic diversity, with multiple sequence types, antimicrobial resistance (AMR) genes, and virulence factors. This study aimed to explore its genomic features and clinical implications. Six clinical isolates from Fujian, Beijing, and Wuhan were sequenced using Illumina and Oxford Nanopore platforms, and antimicrobial susceptibility was tested. Other 139 genomes were retrieved from GenBank. Multi-locus sequence typing, phylogenetic and recombination analyses, resistance/virulence gene detection, plasmid replicon identification, and staphylococcal cassette chromosome *mec* (SCC*mec*) typing were performed. Among 144 genomes, 19 sequence types (STs) were grouped into five clonal complexes (CCs), with CC3 (notably ST3) and ST27 predominating. Eighteen AMR genes were identified, with *blaZ* (37.5%), *qacD* (34.7%), and *mecA* (14.6%) most frequent. Seven isolates carried ≥5 AMR genes. Virulence genes, including cap8E and esxA, were widely distributed. SCC*mec* elements were identified in all *mecA*-positive isolates, with type V predominant in ST3 and type II in ST6. Importantly, 76.2% of SCC*mec*-positive isolates were infection-associated. Novel plasmids carrying multiple resistance genes were identified in two ST3 isolates. This study reveals extensive genetic diversity, widespread AMRs, and virulence determinants in *S. lugdunensis*, providing insights for surveillance and therapy.

**IMPORTANCE:**

This study highlights a direct public health threat: we found that infection-associated strains of *S. lugdunensis* frequently carry antimicrobial resistance genes and virulence factors, underscoring their potential to cause severe, hard-to-treat infections. It provides a genetic foundation for surveillance: the identification of predominant high-risk lineages and novel plasmids carrying multiple resistance genes offers crucial molecular targets for future tracking and monitoring of this emerging pathogen. It informs clinical decision-making: understanding the genetic basis of its resistance and virulence is a critical step toward developing more effective strategies for infection control and guiding targeted antibiotic therapy.

## INTRODUCTION

*Staphylococcus lugdunensis* (*S. lugdunensis*) is a coagulase-negative staphylococcus (CoNS) species initially regarded as a skin commensal, frequently isolated from areas such as the groin, perineum, and axilla (1). However, unlike other CoNS—often dismissed as contaminants or minor pathogens, *S. lugdunensis* has emerged as a clinically opportunistic pathogen capable of causing severe, invasive infections with notable treatment failure rates. Documented infections included infective endocarditis (IE), skin and soft tissue infections (SSTIs), prosthetic joint infections (PJIs), and even bloodstream infections (2–4). Growing evidence revealed that *S. lugdunensis* exhibited an infectious potential and unusually high virulence, more closely resembling *Staphylococcus aureus* (*S. aureus*) than other typically CoNS (4, 5).

Usually, genomic and phylogenetic analyses serve as powerful tools to elucidate the genetic characteristics and population structure of pathogenic species, and to link specific lineages to virulence traits and antimicrobial susceptibility profiles (6, 7). Multi-locus sequence typing (MLST) has generated 20 different sequence types (STs) and five clonal complexes (CCs) of *S. lugdunensis* based on seven housekeeping genes. However, MLST alone has offered limited resolution for pinpointing hypervirulent or multidrug-resistant lineages, particularly among carriage isolates (8).

The advent of whole-genome sequencing (WGS) has revolutionized our understanding of bacterial molecular epidemiology and resistance mechanisms (9, 10). Recent WGS-based investigations have revealed a broader spectrum of virulence determinants and resistant genes in *S. lugdunensis* (11–14). In parallel, mobile genetic elements (MGEs), including plasmids and bacteriophages, have been extensively characterized in *S. aureus* for their roles in horizontal gene transfer, yet their prevalence and diversity in *S. lugdunensis* remain poorly defined (12, 13, 15).

Despite its recognized clinical importance, the global population structure, genomic diversity, and evolutionary dynamics of *S. lugdunensis* are not fully understood. To address these gaps, we performed comprehensive WGS analysis on 6 newly isolated clinical strains from China and integrated these data with 138 publicly available genomes. Our study systematically assesses the distribution of STs, resistance genes, virulence factors, and staphylococcal cassette chromosome *mec* (SCC*mec*) subtypes, thereby providing a holistic genomic framework for this emerging pathogen.

## MATERIALS AND METHODS

### Whole genome sequencing and published data collection

Six clinical isolates from patients were collected from Fujian (4 isolates), Beijing (1 isolate), and Wuhan (1 isolate) (called by RMLUG1-6) in our study. These isolates underwent complete genome sequencing using both short-read sequencing on the Illumina platform and long-read sequencing on the Oxford Nanopore platform, as previously described (16). To investigate the genomic characteristics of *S. lugdunensis*, we also retrieved another 138 published genomes from the GenBank database of National Center for Biotechnology Information (accessed on 26 May 2025) (Table S1). For the isolates that were sequenced multiple times, only the sequence with the highest quality was remained.

### Antimicrobial susceptibility test

Antimicrobial susceptibility of six clinical isolates for 19 antibiotics was determined using the VITEK2 Compact (Biomerieux, France) according to the Clinical and Laboratory Standards Institute (CLSI) guidelines (M100S, 30th edition) (Fig. S1). The minimum inhibitory concentrations (MICs) of oxacillin were tested using the broth microdilution method, with results interpreted according to the CLSI (susceptible ≤2 µg/mL, resistant ≥4 µg/mL). The MIC of cefoxitin was tested using the disc diffusion tests as outlined by the CLSI using Muller-Hinton (MH) agar from Becton Dickinson (BD, USA). The results were interpreted according to the zone diameter (susceptible ≥22 mm, resistant ≤21 mm). The absence and presence of growth (more than 1 colony or a thin film) was considered indicating susceptibility and resistance, respectively. Methicillin resistance was evaluated based on resistance to oxacillin and cefoxitin. *S. aureus* ATCC29213 and ATCC43300 were included for quality control.

### Multi-locus sequence typing and phylogenetic analysis

Molecular typing was performed using MLST scheme for *S. lugdunensis* (https://bigsdb.pasteur.fr/staphlugdunensis/). A minimal spanning tree was constructed based on the allelic profiles of the STs using the goeBURST algorithm in PHYLOViZ v2.0 (http://www.phyloviz.net/) (17). The chromosomal sequence of *S. aureus* USA300 (accession no: NC_010079) was used as the reference for phylogenetic analysis as previously described. Briefly, we compared the *S. lugdunensis* genome sequences to the reference and identified the single nucleotide polymorphisms (SNPs) using MUMmer v3.23 (18). Recombination sites were detected by Gubbins, and the concatenated sequences of filtered polymorphic sites conserved across all genomes were then used to perform phylogenetic analysis by FastTree v2.1.11 (19).

### Detection of sequence types, antimicrobial resistance genes, and virulence factors

Primary gene prediction and annotation were performed using Prokka v1.14 (20). We then identified resistance genes, virulence genes, insertion sequences (IS), and plasmid replicon types by BLAST v2.2.17 compared with ResFinder (21), Virulence Factor Database (VFDB) (22), VirulenceFinder database from Center for Genomic Epidemiology (23), IsFinder (24), and plasmidFinder (25) databases. We also identified plasmid sequences by comparing the genome sequences with NCBI Refseq plasmid database.

### Detection of mobile genetic elements and the pangenome of *S. lugdunensis*

The detection and subtyping of SCC*mec* were performed using sccmec software v1.2.0 (https://github.com/rpetit3/sccmec). Pathogenicity islands (PAIs) were predicted based on the comparison with VFDB, and the regions with at least two VFs located in 10 kbp were considered PAIs. Prophages were identified using PhiSpy v 4.2.21 (26), integrative and conjugative elements (ICEs) were identified using ICEfinder (27), and these MGEs were further clustered using MMseq2 v18.8 (28). The pangenome of *S. lugdunensis* was detected by roary v3.9.1 using the default parameters (29).

## RESULTS

### Origins of *S. lugdunensis* isolates and genomes

**Fig 1:**
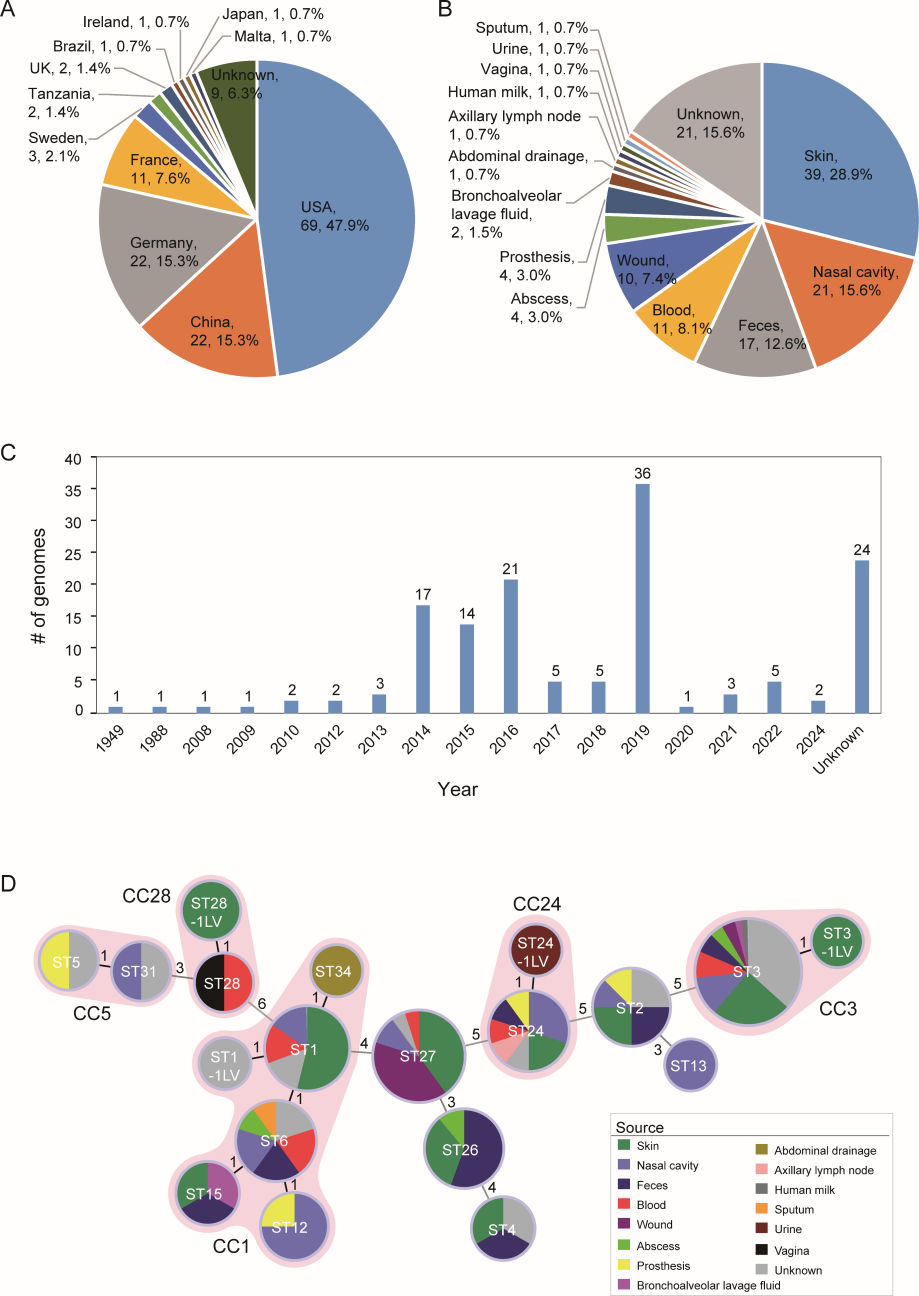
The background information of the 144 *S. lugdunensis* genomes. (**A**) The geographic origin of the genomes. The fans represent the number and proportion of genomes from each country among the 144 genomes. (**B**) The sources of the genomes from human isolates or samples. The fans represent the number and proportion of each sample type among the 135 genomes from human. (**C**) The collection time of the isolates and samples. The histograms represent the number of isolates and samples collected in each year. (**D**) Minimal spanning tree based on the multiple-locus typing results. Each circle represents a ST, and the fans in different colors represent the proportion of genomes from different types of samples among the ST. The number on the line between two circles represents the number of different loci between two STs. The STs that differed by a locus are designated as the same clonal complex (CCs) and marked by light red shadow. Five CCs are identified in total.

In addition to the six complete genomes of clinical isolates sequenced in this study, we collected 138 publicly available genome sequences, including 121 genomes from *S. lugdunensis* isolates and 17 metagenome-assembled genomes directly obtained from feces samples. These isolates and samples originated from a variety of specimen types collected from both patients and healthy individuals across the globe (Fig. 1). Among the 144 genomes in total, 135 had identifiable geographic origins (Fig. 1A), including the United States (69 isolates), China (22), Germany (22), France (11), and seven other countries (11). The majority were derived from human specimens (93.8%, 133/144, Fig. 1B). Two genomes were from environmental sources, while the host information was unavailable for the remaining seven. Most isolates and samples were obtained after the year 2000 (Fig. 1C).

Of the 135 genomes associated with human sources, 114 had known anatomical origins (Fig. 1D). Thirty-one isolates (23.0%) were collected from infection sites or normally sterile body sites and were, thus, considered infection-related. These included isolates from blood (8.1%, 11/135), wound (7.4%, 10/135), abscess (3.0%, 4/135), prosthetic devices (3.0%, 4/135), abdominal drainage (0.7%, 1/135), and axillary lymph node (0.7%, 1/135). More than half of the isolates were deemed to represent colonization, including those from skin (28.9%, 39/135), nasal cavity (15.6%, 21/135), and feces (12.6%, 17/135).

### Molecular typing revealed broad distribution of *S. lugdunensis* subtypes

Molecular typing using MLST identified 19 STs among the 144 *S. lugdunensis* genomes analyzed (Fig. 1D). STs could not be determined for two genomes due to low-quality data at the typing loci. The most common types were ST3 (49 isolates), ST27 (20), ST1 (13), ST24 (12), and ST6 (10). In addition, five clonal complexes (CCs) were identified. CC3 was the largest one, containing 2 STs and 50 genomes, followed by CC1, which included 6 STs and 32 isolates. CC24 included 2 STs and 13 isolates, while both CC5 and CC28 consisted of 2 STs and 4 isolates each.

**Fig 2:**
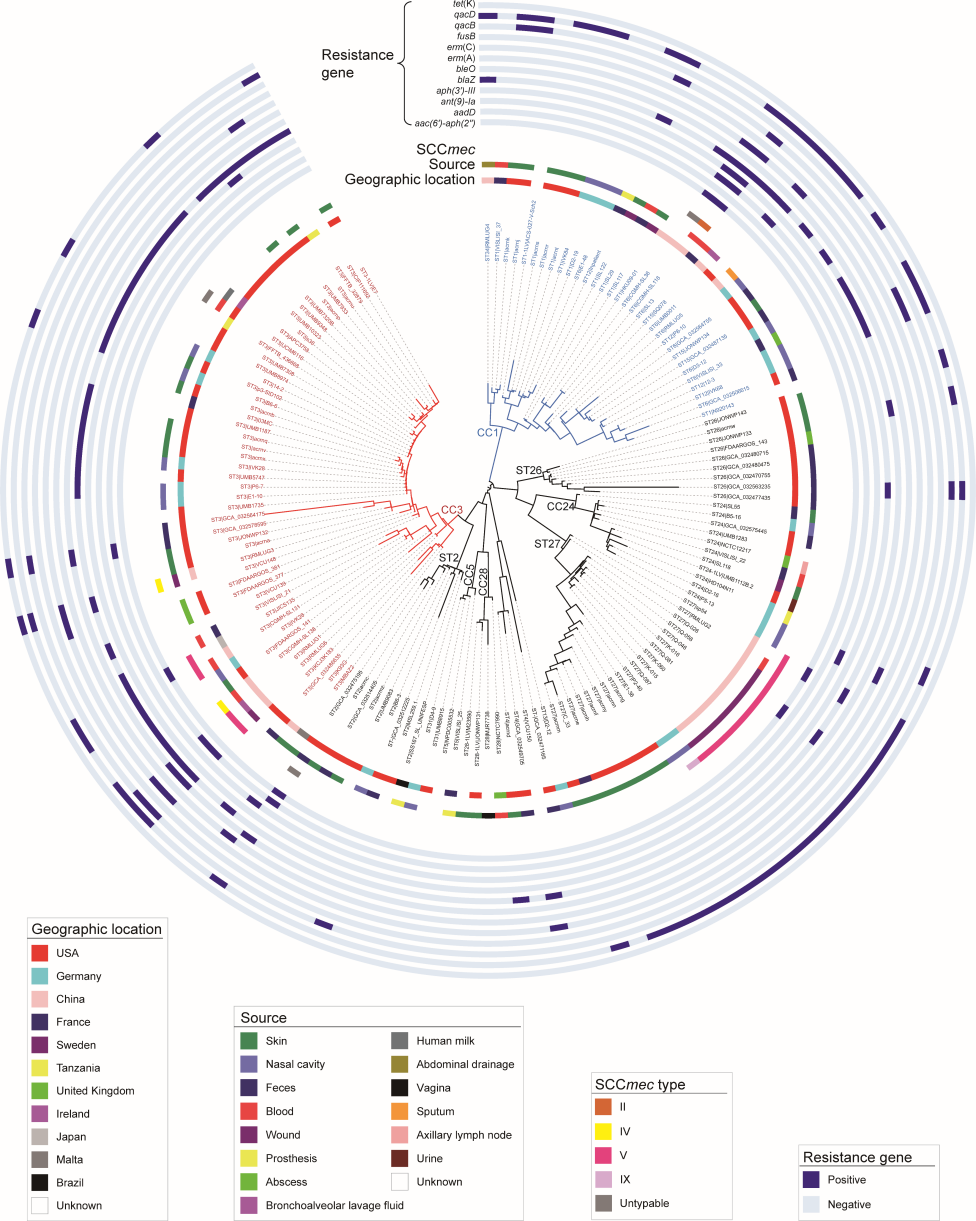
Phylogenetic relationships of the genomes collected in this study. The circular phylogenetic tree of the 144 genomes was reconstructed from whole genome data by the maximum likelihood method. The rings from inside to outside represent the geographic origin, the collection year and sample sources of the isolates, the SCC*mec* subtypes of the SCC*mec*-positive genomes, and the carriage of 12 resistance genes identified among the genomes.

To investigate phylogenetic relationships, we constructed a phylogenetic tree based on core genome single nucleotide polymorphisms (SNPs) identified across the 144 genomes (Fig. 2). The resulting phylogeny was consistent with the MLST classification. Genomes from CC1 were clustered into a distinguished clade (clade 1), those of ST26, CC24, and ST27 were clustered into clade 2, and the others into clade 3. All STs containing more than two isolates were obtained from multiple body sites, indicating widespread distribution (Fig. 1D). Notably, isolates belonging to ST1, ST2, ST3, ST5, ST6, ST12, ST24, ST26, ST27, ST28, and ST34 were related to infections at multiple body sites including blood stream, wound, abscess, and prosthetic devices. These findings suggest that *S. lugdunensis* exhibits broad tissue tropism and that its subtypes lack strong site specificity.

### Antimicrobial resistance genes in *S. lugdunensis* genomes

**Fig 3:**
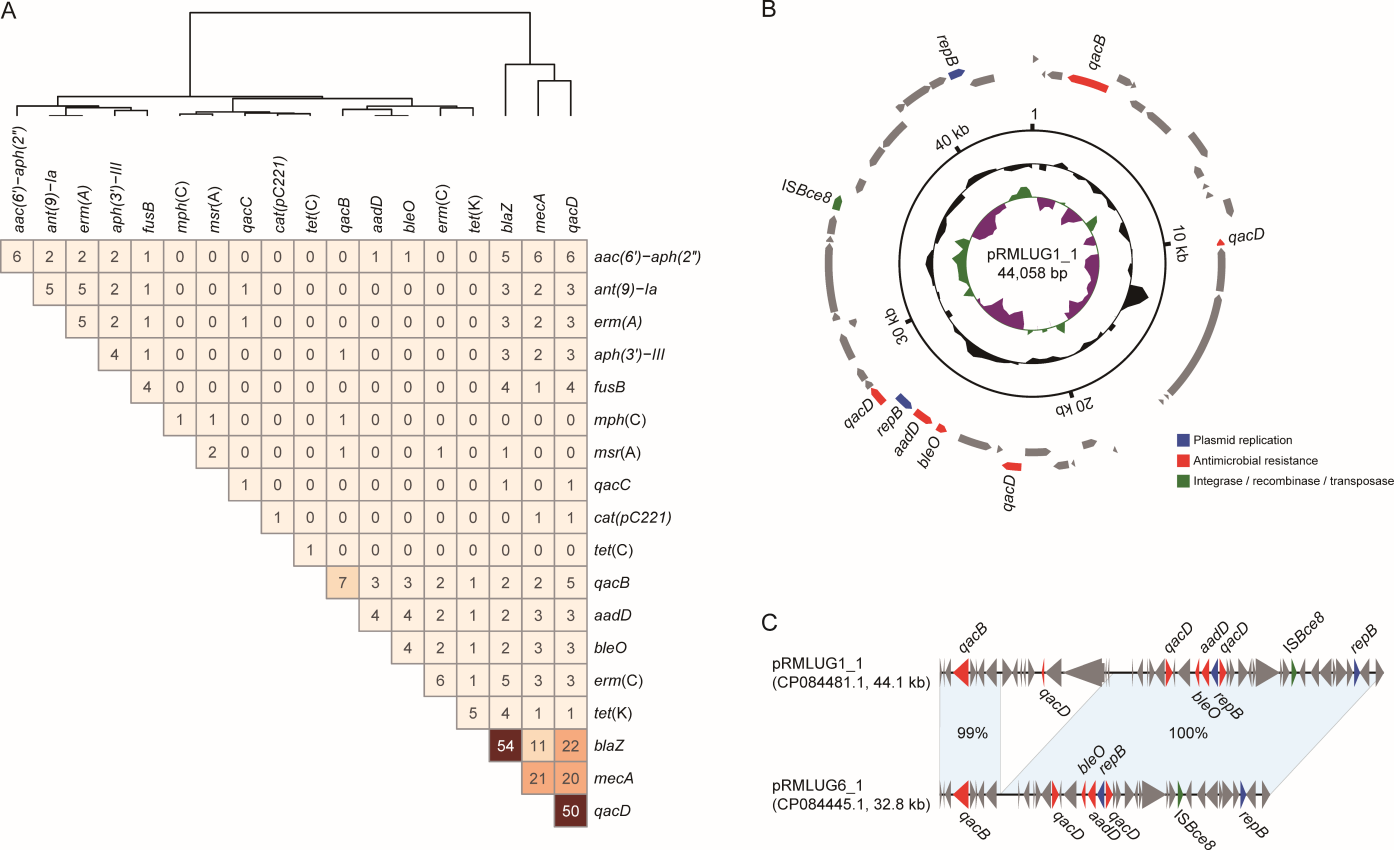
Identification of resistance genes and plasmids. (**A**) The resistance genes identified among the 144 genomes and the co-occurrence of these genes. The numbers on the heatmap represent the number of genomes carrying both the two genes. (**B**) The circular sketch map of the plasmid pRMLUG1_1. The rings from inside to outside represent the GC skew, GC content, genomic coordinate, and predicted genes. The arrows represent the genes related to resistance and transfer (red: resistance; green: integrase recombinase and transposase; dark blue: plasmid replication; gray: other functions). (**C**) The alignment of plasmid pRMLUG6_1 and plasmid pRMLUG1_1. The matched regions between two sequences are displayed by light blue blocks, and the identities are marked.

**Fig 4:**
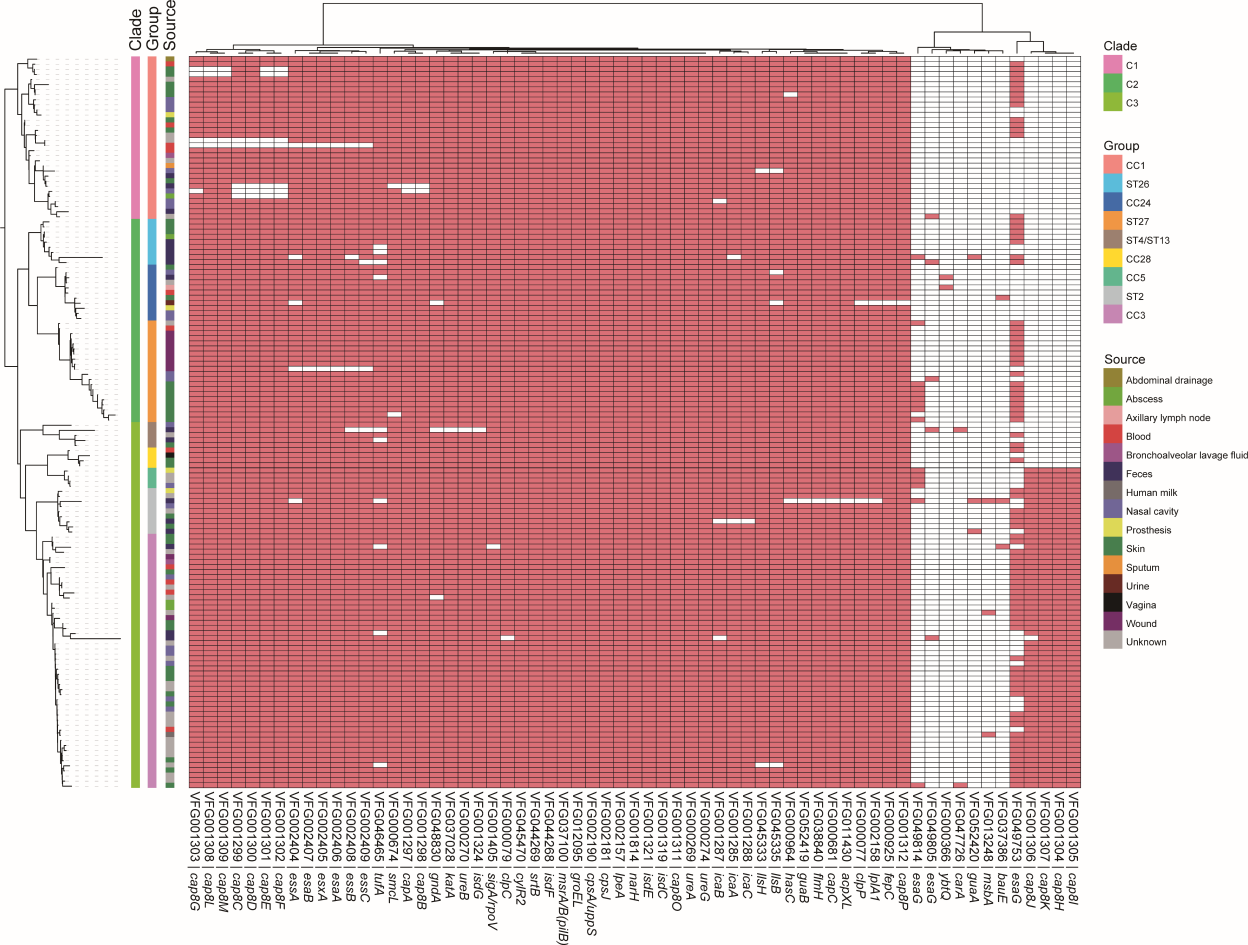
Carriage of virulence genes among the *S. lugdunensis* genomes collected in this study. At the left is the same phylogenetic tree as in Fig. 2. Three large clades and 9 groups of different clonal complex and STs are marked. The heatmap represents the presence (red) or absence (white) of the virulence factors based on comparison with VFDB.

A total of 18 antimicrobial resistance genes were identified across the 144 *S. lugdunensis* genomes, with 13 of them present in more than 2 genomes (Fig. 2 and 3A). The most common resistance genes were *blaZ*, encoding the BlaZ β-lactamase and conferring resistance to penicillin β-lactam (detected in 37.5%, 54 genomes), *qacD*, encoding a quaternary ammonium compound efflux pump conferring resistance to quaternary ammonium compounds like chlorhexidine (34.7%, 50 genomes), and *mecA*, encoding penicillin-binding protein PBP2a and conferring resistance to penicillin β-lactams (14.6%, 21 genomes). These three genes showed high levels of co-occurrence within these genomes (Fig. 4A), suggesting possible co-selection under antimicrobial pressure. Their presence may contribute to the persistence of *S. lugdunensis* in clinical environments. In addition, each genome carried 1–3 resistance genes, and only a small proportion (4.9%, 7/144) harbored 5 or more resistance genes, including 3 ST3 (CC3), 3 ST6 (CC1), and 1 ST2 isolates. These results suggested a potentially higher carriage of resistance genes in lineages CC1 and CC3; however, this trend did not reach statistical significance (*P* > 0.05), possibly due to the limited number of isolates with multiple resistance genes.

Among the six clinical isolates we sequenced, 19 antibiotics were tested (Fig. S1). We found that *blaZ* was detected in RMLUG1, RMLUG3, RMLUG4, and RMLUG6, consistent with their phenotypic resistance to penicillin. Meantime, RMLUG1, RMLUG3, and RMLUG6 were resistant to cefoxitin and oxacillin. Similarly, *erm*(C) was found in RMLUG1, RMLUG3, and RMLUG6, correlating with resistance to erythromycin and clindamycin. However, these three methicillin-resistant strains were susceptible to antibacterial agents showing activity against methicillin-resistant *S. aureus*, such as linezolid, vancomycin, and teicoplanin (Fig. S1).

### Carriage of SCC*mec* and clinical relevance of SCC*mec*-positive isolates

Consistent with the presence of *mecA* gene, all 21 *mecA*-positive genomes were also positive for the SCC*mec* elements, comprising four known SCC*mec* types. For three isolates, the SCC*mec* type could not be determined (Fig. 2). One isolate, RMLUG2, belonged to the ST27 cluster carrying SCC*mec* type V. This cluster comprised 10 isolates originating from China, including isolate sp54, recovered from a swimming pool water sample in Shandong province; RMLUG2, obtained from a patient’s blood sample in Fujian province; and seven isolates derived from wound specimens of patients in Hong Kong. These results indicated widespread dissemination of this ST27 cluster within China. In contrast, another ST27 isolate, P2-40, from Germany, did not cluster with Chinese ST27 group and harbored SCC*mec* type IX.

Among ST3, four isolates carried SCC*mec* type V—including two from our study (RMLUG1 and RMLUG6), while two carried SCC*mec* type IV, including RMLUG3. One additional SCC*mec*-positive ST3 isolate could not be typed. A single ST6 isolate carried SCC*mec* type II. The diversity of SCC*mec* subtypes observed among different sequence types suggests that *S. lugdunensis* has acquired these elements via multiple horizontal gene transfer events.

Clinically, SCC*mec*-positive isolates showed strong associations with infection. Of the 21 SCC*mec*-positive isolates, 16 (76.2%) were from patient specimens, including 10 from wound samples, 5 from blood samples, and 1 from bronchoalveolar lavage fluid sample. Furthermore, among the 31 isolates from infection sites or normally sterile sites, SCC*mec*-positive isolates accounted for a high proportion (48.4%, 15/31), underscoring their potential clinical significance.

### Plasmids carrying resistance genes

We also identified two similar resistance plasmids in the ST3 isolates RMLUG1 and RMLUG6 sequenced in this study. These plasmids represent a novel type, with no closely related plasmids found in the NCBI GenBank database. The plasmid pRMLUG1_1 of isolate RMLUG1 was 44.1 kb in length, carrying several resistance genes including *bleO*, *aadD*, *qacB*, and three copies of *qacD*. The plasmid pRMLUG6_1 of isolate RMLUG6 was 32.8 kb in length, which was highly similar to plasmid pRMLUG1_1 (74.4% coverage and 99% identity), carrying similar resistance genes. However, it lacked the fragment containing the virulence associated gene hostimm-ACME and one copy of *qacD*. Few plasmids were identified among the other genomes, even in the ones with complete sequences.

### Virulence factors among *S. lugdunensis* genomes

Based on the comparison with the experimentally validated VFs from pathogenic bacteria in VFDB, we identified 107 VFs among the 144 genomes in total, of which 63 were presented in at least 2 genomes (Fig. 4). These VFs were mainly related to the synthesis of type 8 capsular polysaccharide (*capA*, *capC*, *cap8B*, *cap8C*, *cap8D*, *cap8E*, *cap8F*, *cap8G*, *cap8H*, *cap8I*, *cap8J*, *cap8K*, *cap8L*, *cap8M*, *cap8O*, *cap8P*), capsule (*cpsA*/*uppS*, *cpsJ*), type VII secretion system (T7SS, *essA*, *essB*, *essC*, *esaA*, *esaB*, *esaG*, *esxA*), polysaccharide intercellular adhesin (PIA) (*icaA*, *icaB*, *icaC*), iron-regulated surface determinant (*isdC*, *isdE*, *isdF*, *isdG*), urease (*ureA*, *ureB*, *ureG*). Other VFs were related to LPS (*acpXL*), ferric siderophore (*bauE*), cytolysin (*cylR2*), ferrienterobactin (*fepC*), lipoprotein promoting cell invasion (*lpeA*), and yersiniabactin (*ybtQ*).

Most of the above VFs were identified in almost all these *S. lugdunensis* genomes. Some VFs were related to specific subtypes and lineages, like *cap8I*, *cap8J*, *cap8K*, and *cap8H* were present in ST2, CC3, and CC5 of clade 3, which might be related to different serotypes. Among three *esaG* paralogs identified, *esaG* (VFG049753) was widely present in CC1, ST26, ST27, ST4, CC28, ST2, and CC3, whereas *esaG* (VFG049814) was mainly present in ST27 and CC5, and the other one (VFG049805) was only found in two genomes. To investigate the relationship between virulence potential and clinical manifestations, we mapped the sample sources onto the phylogenetic tree (Fig. 4). However, no distinct clustering or specific virulence gene patterns were observed for isolates derived from invasive sites (e.g., blood stream) compared to non-sterile sites.

### Identification of MGEs related to pathogenicity and resistance

**Fig 5:**
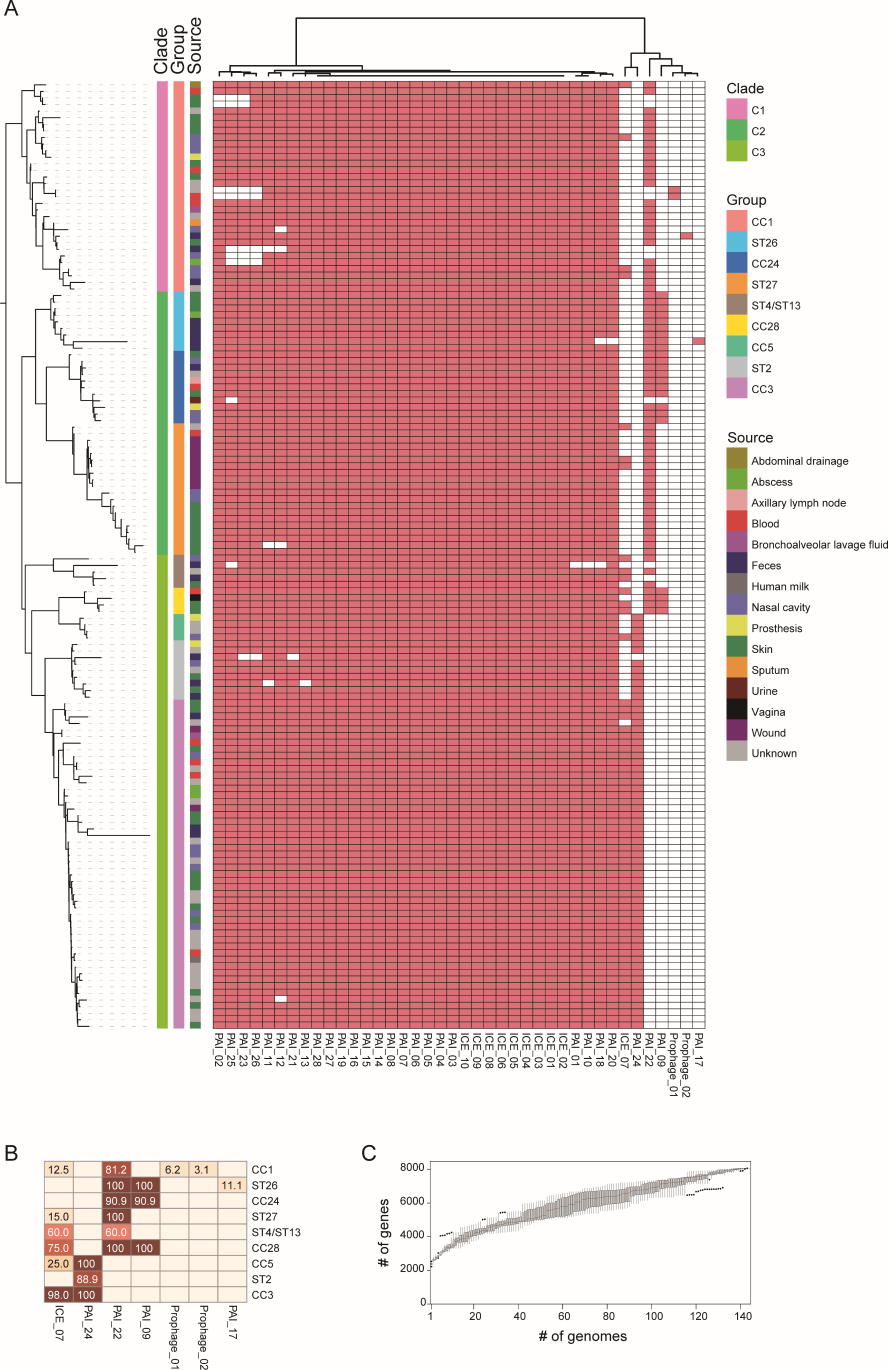
Identification of MGEs including ICEs, prophages, PAIs, and pangenome of *S. lugdunensis*. (**A**) The carriage of MGEs among the 144 genomes. At the left is the same phylogenetic tree as in Fig. 2 and 4. The heatmap represents the presence (red) or absence (white) of the MGEs. (**B**) The carriage rates of seven MGEs which are differentially distributed among the nine groups. The number on the cells of the heatmap represents the carriage rates. (**C**) The increase of the pangenome size represented by the number of unique genes on *y* axis along with the number of genomes on *x* axis.

**Fig 6:**
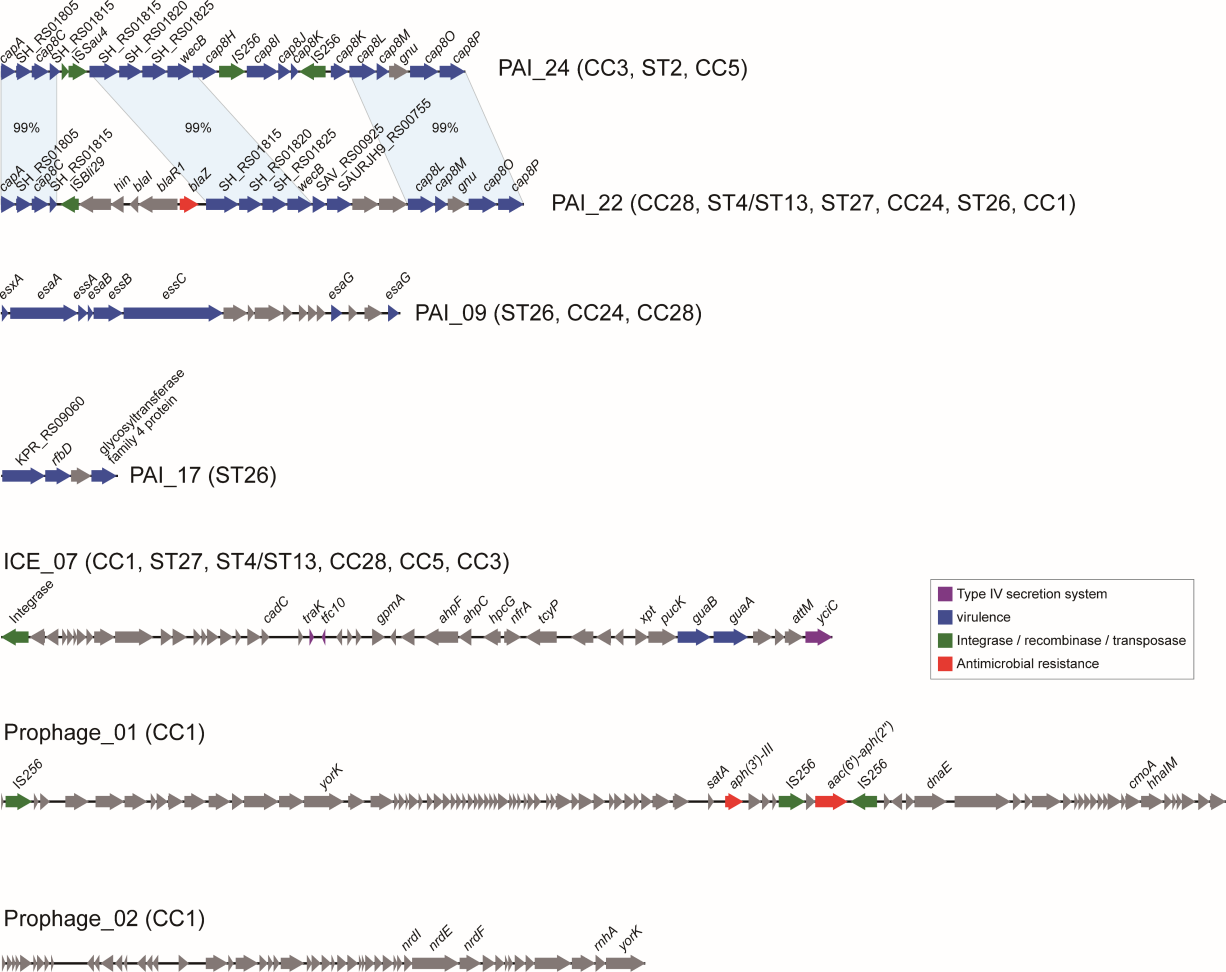
The genetic compositions of the MGEs which are differentially distributed among the nine groups. The arrows represent the genes related to resistance, virulence, and transfer (red: resistance; green: integrase recombinase and transposase; dark blue: virulence; gray: other functions).

Based on the VFs identified, we further detected the VFs located together in the genomes as pathogenicity islands. We also detected ICEs and prophages. Finally, we identified 28 PAIs, 10 ICEs, and 2 prophages among these genomes (Table S2). The composition and distribution of PAIs were similar to the characteristics of VFs (Fig. 5A). Most of the PAIs were present in all *S. lugdunensis* subtypes, and some were differentially distributed. Two clusters (PAI_22 and PAI_24) were comprised of different genes encoding type 8 capsular polysaccharide (Fig. 6). PAI_24 was present in ST2, CC3, and CC5, whereas PAI_22 was present in other subtypes. Three conserved regions with 99% identity were identified, and a transposon containing resistance gene *blaZ* and IS*Bli29* was found in PAI_22. PAI_09 comprised genes of T7SS, and the entire islands were present in ST26, CC24, and CC28, whereas partial sequences lacking the *esaG* were present in other subtypes. Among the 10 ICEs, 9 were found in all the genomes, and ICE_07 was found in the groups of CC1, ST27, ST4/ST13, CC28, CC5, and CC3 with different carriage rates. Genes encoding integrases and type IV secretion system were identified in these ICEs. Two prophages, Prophage_01 was present in two genomes of CC1, and a transposon containing resistance genes *aph(3')-III* and *aac(6')-aph(2''*), and two copies of IS*256* were identified. The other prophage was present in only one genome of CC1 and contained the *nrdI*, *nrdE,* and *nrdF* genes mediating tolerance to oxidative stress. The number of unique genes in the pangenome was continually increasing along with the increase of genome numbers, indicating that we cannot obtain a closed pangenome of *S. lugdunensis* based on these data, which is in line with the high diversity reflected by the MLST and phylogenetic studies.

## DISCUSSION

*S. lugdunensis*, a CoNS first described in 1988, is part of the normal human skin flora but emerged as a significant human pathogen gradually (30). This study presents a global genomic analysis of 144 *S. lugdunensis* isolates from clinical and environmental sources, revealing substantial genetic diversity (19 STs and CCs), with ST3 and ST27 being predominant in Western and Asian regions, respectively.

Although traditional antimicrobial susceptibility surveillance showed that most *S. lugdunensis* isolates remain susceptible to beta-lactams, an increasing number of oxacillin-resistant *S. lugdunensis* (ORSL) strains are being reported in hospital settings, particularly in Asia (12, 31). In our study, approximately 14.6% of isolates carried SCC*mec* elements, primarily types IV and V. Among them, 10 isolates carrying SCC*mec* V belonged to the ST27 cluster from China. However, one of these (RMLUG2) remained phenotypically susceptible to oxacillin. These findings align with prior observations by Chang et al., who reported that ST27 strains (e.g., SL29 and SL35) frequently harbor SCC*mec* V yet retain oxacillin susceptibility, whereas strains such as SL149, carrying SCC*mec* Vt, display resistance (11). Ho et al. emphasized that the current disk and MIC-based breakpoints may not reliably detect oxacillin resistance in certain lineages, particularly ST27 (32).

Besides, RMLUG1 and RMLUG6 (ST3) carried SCC*mec* V, while RMLUG3 (also ST3) harbored SCC*mec* IV. All three were phenotypically oxacillin-resistant. Meanwhile, only one ST6 isolate carried SCC*mec* II. While SCC*mec* V has been identified as the most common type in ORSL strains (33), SCC*mec* II–ST6 associations have been implicated in hospital endemic transmission in Taiwan (12, 34, 35). But, Tein-Yao Chang also documented oxacillin-susceptible ST6 strains carrying SCCmec II (11). These inconsistencies between SCC*mec* carriage and oxacillin susceptibility may stem from differences in susceptibility testing methodologies, SCC*mec* subtypes, and regulatory mechanisms governing *mec* gene expression.

Beyond SCC*mec*, we identified 18 AMR genes across our genome collection. The β-lactamase gene *blaZ* was the most prevalent, detected in over one-third of genomes. Among our six clinical isolates, penicillin resistance was fully concordant with blaZ presence, in agreement with previous studies (36), suggesting widespread resistance to penicillin-class antibiotics in *S. lugdunensis*. A subset (4.9%) of isolates carried five or more AMR genes, including three from ST3 (CC3). Two ST3 strains (RMLUG1 and RMLUG6) also carried novel plasmids: pRMLUG1_1 encoded multiple resistance determinants (*bleO*, *aadD*, *qacB*, and three copies of *qacD*), while pRMLUG6_1 shared similar features but lacked one *qacD* copy. Chang et al. have suggested that ST3 strains lack CRISPR-Cas systems and exhibit greater SCC*mec* diversity, potentially facilitating horizontal gene transfer and the acquisition of resistance elements (11). However, the remaining 138 genomes were retrieved from public databases, where associated phenotypic data were either limited or unavailable. Due to this lack of comprehensive phenotypic information for most of the data set, a robust statistical correlation between antimicrobial resistance genotypes and phenotypes could not be fully established in this study. Consequently, the precise relationship between specific resistance genes and phenotypic outcomes in *S. lugdunensis* warrants further exploration and validation in future studies with larger cohorts of paired genomic and phenotypic data.

The genomic landscape of *S. lugdunensis* analyzed in this study reveals a rich repertoire of virulence-associated genes, providing a molecular rationale for its pathogenic potential. Our screening of 144 genomes identified 107 putative VFs, indicating that this species possesses a comprehensive genomic framework for colonization and host interaction, different from other CoNS (4). Several key factors have been implicated, including adherence and biofilm formation, iron acquisition systems, immune evasion and capsule, secretion system, enzymes, toxins, and so on. A consistent feature across the studied genomes is the ubiquitous presence of genes predicted to be involved in adhesion and nutrient acquisition. Specifically, the ica operon (icaA, icaB, icaC), which encodes the enzymes required for polysaccharide intercellular adhesin (PIA) synthesis, was detected in nearly all isolates (Fig. 4). The widespread conservation of these genes may suggest a universal genomic capacity for polysaccharide-mediated biofilm formation, a possible virulence mechanism in staphylococcal infections (37). Furthermore, the complete isd gene cluster (isdC, isdE, isdF, isdG), responsible for iron-regulated surface determinants, was identified throughout the population. The presence of this locus, which is homologous to the iron-acquisition system in *S. aureus* but typically absent in *S. epidermidis*, implies a genetically encoded potential for heme-iron utilization, a critical trait for survival in iron-restricted host environments (38).

Beyond the conserved core, our analysis revealed notable genetic diversity in loci associated with surface structures and secretion systems. The type VIII capsular polysaccharide gene cluster (cap8) exhibited lineage-dependent distribution patterns, with specific genes such as cap8I, cap8J, cap8K, and cap8H being exclusively associated with Clade 3 (ST2, CC3, and CC5). This genomic variation suggests that different lineages may encode distinct capsular structures, pointing to a genetic basis for potential serotypic diversity (14). Similarly, the Type VII secretion system (T7SS) displayed significant variation. We identified multiple paralogs of the esaG gene, with distinct variants segregating according to clonal complex (e.g., VFG049753 in CC1/ST26 vs VFG049814 in ST27/CC5), which may imply different repertoires of secretion effectors. Our findings align with previous studies suggesting that *S. lugdunensis* possesses a conserved core virulome. The high prevalence of these genes across all anatomical sources indicates that the transition from commensalism to invasive infection may be driven by host susceptibility and environmental cues rather than the acquisition of specific virulence determinants.

Despite the insights provided, this study has several limitations. First, we were unable to fully investigate the discordance between SCC*mec* presence and oxacillin resistance due to limited phenotypic data. More accurate and standardized susceptibility testing methods are needed, and future studies should include larger numbers of isolates with detailed resistance profiles. Second, the functional validation of putative virulence factors—including their expression, regulatory pathways, and contributions to pathogenesis—was not performed, limiting the conclusions that can be drawn regarding their clinical significance.

This study represents one of the most comprehensive genomic investigations of *S. lugdunensis* to date and, for the first time, identifies novel ST3 plasmids co-harboring resistance and virulence genes alongside diverse SCC*mec* subtypes linked to clinical infections. By leveraging WGS, we provide an in-depth genome characterization of *S. lugdunensis*. Our findings suggest that *S. lugdunensis* may harbor a wider array of resistance genes than previously recognized, laying the foundation for targeted surveillance.

## Data Availability

The sequencing data generated in this study have been deposited in the National Center for Biotechnology Information (NCBI) database under the accession number PRJNA763873.

## SUPPLEMENTAL MATERIAL

Supplemental material aem.01893-25-s0001.docx Fig S1; Tables S1 and S2.

ASM does not own the copyrights to Supplemental Material that may be linked to, or accessed through, an article. The authors have granted ASM a non-exclusive, world-wide license to publish the Supplemental Material files. Please contact the corresponding author directly for reuse.
