# Supplementary material for: Global genomic landscape of Staphylococcus lugdunensis: population structure, antimicrobial resistance, and virulence determinants
Source: Appl Environ Microbiol. 2026 Feb 23;92(3):e01893-25. doi: 10.1128/aem.01893-25 (PMC12997805; doi:10.1128/aem.01893-25)
Supplement: Supplemental material — Fig S1; Tables S1 and S2. [file aem.01893-25-s0001.docx]

**Supplementary materials**

**Contents of Supplements**

[Fig S1 The antimicrobial susceptibility results and genes of the six clinical isolates we sequenced 2](#_Toc221990915)

[Table S1 Metadata of the *Staphylococcus lugdunensis* genomes 3](#_Toc221990916)

[Table S2 MGEs identified among the *Staphylococcus lugdunensis* genomes 13](#_Toc221990917)

# Fig S1 The antimicrobial susceptibility results and genes of the six clinical isolates we sequenced


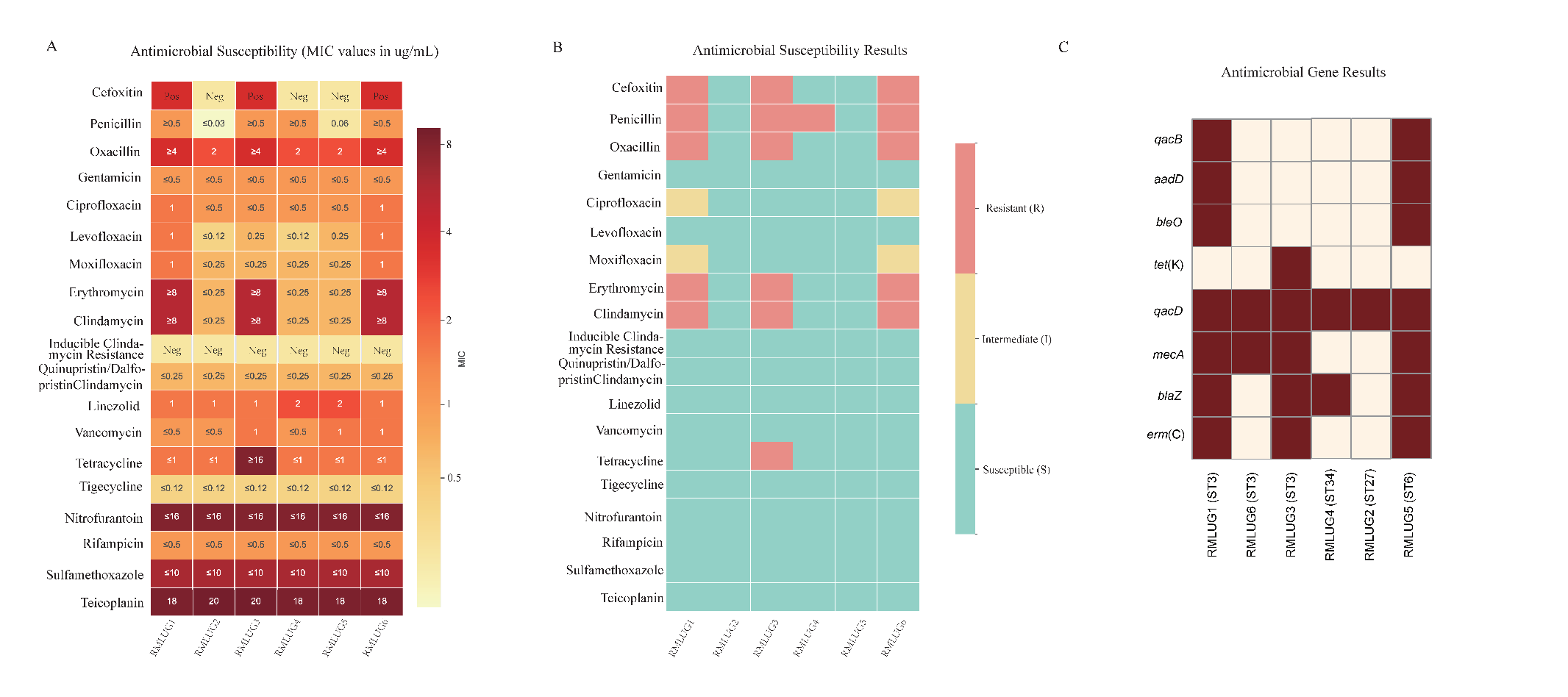


The MIC values (A), antimicrobial susceptibility results (B), and the antimicrobial genes (C) of six clinical isolates we sequenced

# Table S1 Metadata of the *Staphylococcus lugdunensis* genomes

| **Accession number** | **Strain** | **Geographic location** | **Collection year** | **Host** | **Source** |
| --- | --- | --- | --- | --- | --- |
| GCA_000025085 | HKU09-01 | China: Hong Kong | - | Human | - |
| GCA_000185485 | M23590 | - | - | Human | Skin |
| GCA_000247225 | VCU139 | - | - | - | - |
| GCA_000270465 | N920143 | - | - | - | - |
| GCA_000316075 | ACS-027-V-Sch2 | - | - | Human | - |
| GCA_000542395 | UCIM6116 | USA | - | Human | Blood |
| GCA_000649085 | VCU150 | - | - | - | - |
| GCA_000649125 | VCU148 | - | - | - | - |
| GCA_001546615 | MJR7738 | - | - | Human | Vagina |
| GCA_001558775 | FDAARGOS_141 | USA | 2014 | Human | Skin |
| GCA_001558815 | FDAARGOS_143 | USA | 2014 | Human | Abscess |
| GCA_002096075 | VISLISI_37 | France | 2015 | Human | Blood |
| GCA_002096115 | VISLISI_21 | France | 2015 | Human | Blood |
| GCA_002096135 | VISLISI_25 | France | 2015 | Human | Prosthesis |
| GCA_002096155 | Inpatient | France | 2014 | Human | Prosthesis |
| GCA_002096975 | C_33 | France | 2015 | Human | Skin |
| GCA_002097035 | VISLISI_22 | France | 2015 | Human | Blood |
| GCA_002104555 | VISLISI_33 | France | 2015 | Human | Abscess |
| GCA_002250095 | K93G | China: Hong Kong | 2014 | Human | Skin |
| GCA_002407165 | FDAARGOS_377 | USA | 2015 | Human | Abscess |
| GCA_002591215 | FDAARGOS_381 | USA | 2015 | Human | Abscess |
| GCA_900478255 | NCTC12217 | United Kingdom | 1988 | Human | Axillary lymph node |
| GCA_004329485 | E7 | USA | 2017 | Human | Skin |
| GCA_008728715 | SL29 | France | - | Human | Blood |
| GCA_008728735 | SL55 | France | - | Human | Skin |
| GCA_008728755 | SL117 | Sweden | - | Human | Skin |
| GCA_008728775 | SL118 | Sweden | - | Human | Skin |
| GCA_008728795 | SL122 | Sweden | - | Human | Skin |
| GCA_008728815 | SL13 | France | - | Human | Blood |
| GCA_009931395 | APC3758 | Ireland | 2017 | Human | Human milk |
| GCA_011403135 | JICS135 | Japan | 2014 | Human | - |
| GCA_014962425 | IVK28 | Germany | 2009 | Human | Nasal cavity |
| GCA_016804245 | MBAZ2 | Malta | 2019 | Human | Skin |
| GCA_016808995 | s38 | USA | 2014 | - | - |
| GCA_016904985 | HD104N11 | Germany | 2018 | Human | Prosthesis |
| GCA_020682785 | SS167_SL_UNIFESP | Brazil | - | Human | Prosthesis |
| GCA_020882195 | CGMH-SL118 | China: Taiwan | 2013 | Human | Blood |
| GCA_022568915 | P5-13 | Germany | 2016 | Human | Nasal cavity |
| GCA_022568935 | D2-12 | Germany | 2016 | Human | Nasal cavity |
| GCA_022568955 | P8-10 | Germany | 2016 | Human | Nasal cavity |
| GCA_022568975 | E1-48 | Germany | 2016 | Human | Nasal cavity |
| GCA_022568985 | P6-7 | Germany | 2016 | Human | Nasal cavity |
| GCA_022569015 | B6-6 | Germany | 2016 | Human | Nasal cavity |
| GCA_022569035 | B6-3 | Germany | 2016 | Human | Nasal cavity |
| GCA_022569055 | B5-16 | Germany | 2016 | Human | Nasal cavity |
| GCA_022569075 | D2-19 | Germany | 2016 | Human | Nasal cavity |
| GCA_022569085 | D3-12 | Germany | 2016 | Human | Nasal cavity |
| GCA_022569095 | D4-9 | Germany | 2016 | Human | Nasal cavity |
| GCA_022569135 | E1-36 | Germany | 2016 | Human | Nasal cavity |
| GCA_022569155 | P2-40 | Germany | 2016 | Human | Nasal cavity |
| GCA_022569175 | IVK84 | Germany | 2016 | Human | Nasal cavity |
| GCA_022569195 | IVK68 | Germany | 2016 | Human | Nasal cavity |
| GCA_022569205 | D2-16 | Germany | 2016 | Human | Nasal cavity |
| GCA_022569235 | IVK28 | Germany | 2016 | Human | Nasal cavity |
| GCA_022569245 | 14-2 | Germany | 2016 | Human | Nasal cavity |
| GCA_022569275 | 12-3 | Germany | 2016 | Human | Nasal cavity |
| GCA_022660955 | E1-10 | Germany | 2016 | Human | Nasal cavity |
| GCA_022689125 | acrnn | USA | 2019 | Human | Skin |
| GCA_022689145 | acrnq | USA | 2019 | Human | Skin |
| GCA_022689165 | acrnm | USA | 2019 | Human | Skin |
| GCA_022689185 | acrnx | USA | 2019 | Human | Skin |
| GCA_022689205 | acrnv | USA | 2019 | Human | Skin |
| GCA_022689225 | acrnu | USA | 2019 | Human | Skin |
| GCA_022689235 | acrnk | USA | 2019 | Human | Skin |
| GCA_022689255 | acrnt | USA | 2019 | Human | Skin |
| GCA_022689285 | acrnh | USA | 2019 | Human | Skin |
| GCA_022689305 | acrnp | USA | 2019 | Human | Skin |
| GCA_022689325 | acrnj | USA | 2019 | Human | Skin |
| GCA_022689335 | acrnw | USA | 2019 | Human | Skin |
| GCA_022689365 | acrnr | USA | 2019 | Human | Skin |
| GCA_022689385 | acrns | USA | 2019 | Human | Skin |
| GCA_022689405 | acrng | USA | 2019 | Human | Skin |
| GCA_022689425 | acrnf | USA | 2019 | Human | Skin |
| GCA_022689445 | acrny | USA | 2019 | Human | Skin |
| GCA_022689465 | acrne | USA | 2019 | Human | Skin |
| GCA_022689485 | acrna | USA | 2019 | Human | Skin |
| GCA_022689495 | acrnc | USA | 2019 | Human | Skin |
| GCA_022689525 | acrnb | USA | 2019 | Human | Skin |
| GCA_022689545 | acrmz | USA | 2019 | Human | Skin |
| GCA_022689605 | acrnd | USA | 2019 | Human | Skin |
| GCA_023508855 | CGMH-SL131 | China: Taiwan | 2014 | Human | Blood |
| GCA_023508875 | CGMH-SL138 | China: Taiwan | 2012 | Human | Blood |
| GCA_023712745 | MSL259.1 | USA | 2008 | Environment | - |
| GCA_024104375 | Q-087 | China: Hong Kong | 2019 | Human | Wound |
| GCA_024104395 | Q-081 | China: Hong Kong | 2019 | Human | Wound |
| GCA_024104415 | Q-046 | China: Hong Kong | 2019 | Human | Wound |
| GCA_024104435 | Q-058 | China: Hong Kong | 2019 | Human | Wound |
| GCA_024104455 | Q-026 | China: Hong Kong | 2019 | Human | Wound |
| GCA_024104475 | K-015 | China: Hong Kong | 2019 | Human | Wound |
| GCA_024104495 | K-060 | China: Hong Kong | 2019 | Human | Wound |
| GCA_024104505 | K-016 | China: Hong Kong | 2019 | Human | Wound |
| GCA_024106275 | KCJ3K183 | USA | 2019 | Human | - |
| GCA_025152445 | p3-SID102 | USA | 2018 | Human | Skin |
| GCA_026427435 | RMLUG1 | China: Fujian | 2019 | Human | Bronchoalveolar lavage fluid |
| GCA_026427455 | RMLUG2 | China: Fujian | 2018 | Human | Blood |
| GCA_026427475 | RMLUG3 | China: Fujian | 2017 | Human | Wound |
| GCA_026427495 | RMLUG4 | China: Fujian | 2017 | Human | Abdominal drainage |
| GCA_026427515 | RMLUG5 | China: Beijing | 2020 | Human | Sputum |
| GCA_026427535 | RMLUG6 | China: Wuhan | 2021 | Human | Wound |
| GCA_030212975 | UMB9348 | USA | 2019 | Human | - |
| GCA_030224835 | UMB1283 | USA | - | Human | - |
| GCA_030225045 | UMB1187 | USA | - | Human | - |
| GCA_030225285 | UMB0011 | USA | - | Human | - |
| GCA_030228425 | UMB1112B.2 | USA | 2015 | Human | Urine |
| GCA_030228685 | UMB10323 | USA | 2019 | Human | - |
| GCA_030230405 | UMB7933 | USA | - | Human | - |
| GCA_032470755 | N4_207_000G1_dasN4_207_000G1_abawaca.59 | USA | 2014 | Human | Feces |
| GCA_032471165 | N4_206_000G1_dasN4_206_000G1_concoct_30 | USA | 2014 | Human | Feces |
| GCA_032475195 | N4_155_006G1_dasN4_155_006G1_abawaca.29 | USA | 2014 | Human | Feces |
| GCA_032477435 | N4_139_000G1_dasN4_139_000G1_maxbin2.maxbin.003 | USA | 2014 | Human | Feces |
| GCA_032480475 | N5_234_000G1_dasN5_234_000G1_abawaca.28 | USA | 2015 | Human | Feces |
| GCA_032480715 | N5_233_013G2_dasN5_233_013G2_abawaca.11 | USA | 2015 | Human | Feces |
| GCA_032486635 | N4_210_000G1_dasN4_210_000G1_abawaca.21 | USA | 2014 | Human | Feces |
| GCA_032487135 | N4_129_000G1_dasN4_129_000G1_concoct_34 | USA | 2014 | Human | Feces |
| GCA_032506615 | N5_256_000G1_dasN5_256_000G1_concoct_30 | USA | 2015 | Human | Feces |
| GCA_032512225 | S2_002_011G1_dasS2_002_011G1_maxbin2.maxbin.003 | USA | 2013 | Human | Feces |
| GCA_032514405 | N5_279_041G1_dasN5_279_041G1_maxbin2.maxbin.006 | USA | 2015 | Human | Feces |
| GCA_032549705 | N1_009_006G1_dasN1_009_006G1_abawaca.15 | USA | 2012 | Human | Feces |
| GCA_032563235 | N4_126_007G1_dasN4_126_007G1_maxbin2.maxbin.004 | USA | 2014 | Human | Feces |
| GCA_032564175 | N4_124_007G1_dasN4_124_007G1_abawaca.30 | USA | 2014 | Human | Feces |
| GCA_032564755 | N4_116_010G1_dasN4_116_010G1_abawaca.13 | USA | 2014 | Human | Feces |
| GCA_032575445 | SP_CRL_000G1_dasSP_CRL_000G1_abawaca.15 | USA | 2010 | Human | Feces |
| GCA_032578595 | S2_012_000G1_dasS2_012_000G1_abawaca.25 | USA | 2014 | Human | Feces |
| GCA_037694565 | JONWP143 | USA | 2022 | Human | Skin |
| GCA_037694975 | JONWP134 | USA | 2022 | Human | Skin |
| GCA_037695005 | JONWP133 | USA | 2022 | Human | Skin |
| GCA_037700005 | JONWP132 | USA | 2022 | Human | Skin |
| GCA_037700085 | JONWP131 | USA | 2022 | Human | Skin |
| GCA_038738095 | UMB8915 | USA | 2018 | Human | - |
| GCA_038738135 | UMB7308 | USA | 2017 | Human | - |
| GCA_038738185 | UMB1735 | USA | 2015 | Human | - |
| GCA_038738265 | UMB5747 | USA | 2016 | Human | - |
| GCA_038738635 | UMB8974 | USA | 2018 | Human | - |
| GCA_039700225 | SQ078 | China: Jinan | 2024 | Human | Bronchoalveolar lavage fluid |
| GCA_040717915 | UMB7320B | USA | - | Human | - |
| GCA_043550065 | CGMH-SL36 | China: Taiwan | 2010 | Human | - |
| GCA_044003545 | UMB9083 | USA | - | Human | - |
| GCA_048144765 | 03MC | France | 2013 | Human | - |
| GCA_049368035 | sp54 | China: Shandong | 2024 | Environment | - |
| GCA_050275335 | NPDC005532 | - | - | - | - |
| GCA_900474705 | NCTC7990 | United Kingdom | 1949 | Human | Blood |
| GCA_964211005 | FFTB_436668 | Tanzania | 2021 | Human | - |
| GCA_964211535 | FFTB_32879 | Tanzania | 2021 | Human | - |
| GCA_965137065 | CIP111692 | - | - | - | - |

# Table S2 MGEs identified among the *Staphylococcus lugdunensis* genomes

| ID | Category | Genome accession | Contig | Start | End | Length | Genes | # of related isolates | Related groups | Related functions |
| --- | --- | --- | --- | --- | --- | --- | --- | --- | --- | --- |
| GCA_030228685_56-606-6043 | PAI_01 | GCA_030228685 | GCA_030228685_56 | 606 | 6043 | 5,438 | aaa, aae | 143 | all | autolysin/adhesin |
| GCA_032549705_30-15789-20940 | PAI_02 | GCA_032549705 | GCA_032549705_30 | 15789 | 20940 | 5,152 | cap8L, cap8M, cap8O, cap8P | 140 | all | type 8 capsular polysaccharide synthesis |
| GCA_000542395_9-24468-42299 | PAI_03 | GCA_000542395 | GCA_000542395_9 | 24468 | 42299 | 17,832 | clpP, eno, lgt, plr/gapA | 144 | all | ATP-dependent Clp protease, |
| GCA_037700085_10-1481-9265 | PAI_04 | GCA_037700085 | GCA_037700085_10 | 1481 | 9265 | 7,785 | clpP, lgt | 144 | all |  |
| GCA_032563235_90-1395-6699 | PAI_05 | GCA_032563235 | GCA_032563235_90 | 1395 | 6699 | 5,305 | clpP, plr/gapA | 144 | all |  |
| GCA_022689325_20-42323-50095 | PAI_06 | GCA_022689325 | GCA_022689325_20 | 42323 | 50095 | 7,773 | cpsJ, lpeA | 144 | all | lipoprotein promoting cell invasion |
| GCA_000247225_34-134802-142469 | PAI_07 | GCA_000247225 | GCA_000247225_34 | 134802 | 142469 | 7,668 | ctpV, oatA | 144 | all |  |
| GCA_037700085_4-2448-8640 | PAI_08 | GCA_037700085 | GCA_037700085_4 | 2448 | 8640 | 6,193 | eno, plr/gapA | 144 | all |  |
| GCA_900474705_1-2005090-2022765 | PAI_09 | GCA_900474705 | GCA_900474705_1 | 2005090 | 2022765 | 17,676 | esaA, esaB, esaG, essA, essB, essC, esxA | 23 | ST26, CC24, CC28 | type VII secretion system |
| GCA_032506615_27-8-8221 | PAI_10 | GCA_032506615 | GCA_032506615_27 | 8 | 8221 | 8,214 | glnA1, nuc | 143 | all |  |
| GCA_038738095_2-498784-518135 | PAI_11 | GCA_038738095 | GCA_038738095_2 | 498784 | 518135 | 19,352 | hlb, icaA, icaB, icaC, llsB, llsH | 141 | all | beta-hemolysin, intercellular adhesion |
| GCA_020682785_1-2426-11831 | PAI_12 | GCA_020682785 | GCA_020682785_1 | 2426 | 11831 | 9,406 | hlb, llsB, llsH | 140 | all | beta-hemolysin |
| GCA_032471165_19-1063-7033 | PAI_13 | GCA_032471165 | GCA_032471165_19 | 1063 | 7033 | 5,971 | icaA, icaB, icaC, lisK | 143 | all | intercellular adhesion |
| GCA_037695005_278-90754-106617 | PAI_14 | GCA_037695005 | GCA_037695005_278 | 90754 | 106617 | 15,864 | isdC, isdE, isdF, isdG, sirA, sirB, sirC, srtB, sugC | 144 | all | iron-regulated surface determinant protein, staphyloferrin B ABC transporter SirABC |
| GCA_032512225_365-3324-9125 | PAI_15 | GCA_032512225 | GCA_032512225_365 | 3324 | 9125 | 5,802 | isdC, isdE, isdF, isdG, srtB | 144 | all | iron-regulated surface determinant protein |
| GCA_030228425_85-1155-6924 | PAI_16 | GCA_030228425 | GCA_030228425_85 | 1155 | 6924 | 5,770 | isdG, sirA, sirB, sirC, srtB | 144 | all | iron-regulated surface determinant protein, staphyloferrin B ABC transporter SirABC |
| GCA_032563235_120-145-5234 | PAI_17 | GCA_032563235 | GCA_032563235_120 | 145 | 5234 | 5,090 | KOX_RS24925, KPR_RS09060, rfbD | 1 | ST26 | LPS |
| GCA_022569095_2-766438-772883 | PAI_18 | GCA_022569095 | GCA_022569095_2 | 766438 | 772883 | 6,446 | lisR, msrA/B(pilB) | 142 | all |  |
| GCA_022569075_2-802082-812101 | PAI_19 | GCA_022569075 | GCA_022569075_2 | 802082 | 812101 | 10,020 | msbA, ureA, ureB, ureG | 144 | all | urease |
| GCA_032506615_19-7905-13131 | PAI_20 | GCA_032506615 | GCA_032506615_19 | 7905 | 13131 | 5,227 | narG, narH | 143 | all | nitrate reductase |
| GCA_032575445_34-19194-24196 | PAI_21 | GCA_032575445 | GCA_032575445_34 | 19194 | 24196 | 5,003 | PMI_RS12860, aae | 143 | all | autolysin/adhesin |
| GCA_039700225_1-358456-381712 | PAI_22 | GCA_039700225 | GCA_039700225_1 | 358456 | 381712 | 23,257 | SAURJH9_RS00755, SAV_RS00925, SH_RS01805, SH_RS01810, SH_RS01815, SH_RS01820, SH_RS01825, SSP_RS00305, cap8L, cap8M, cap8O, cap8P, wecB | 72 | CC28, ST4/ST13, ST27, CC24, ST26, CC1 | type 8 capsular polysaccharide synthesis |
| GCA_032477435_74-6368-12493 | PAI_23 | GCA_032477435 | GCA_032477435_74 | 6368 | 12493 | 6,126 | SH_RS01805, SH_RS01810, SH_RS01815, SH_RS01820, SH_RS01825, SSP_RS00305 | 136 | all | type 8 capsular polysaccharide synthesis |
| GCA_011403135_1-420978-441629 | PAI_24 | GCA_011403135 | GCA_011403135_1 | 420978 | 441629 | 20,652 | SH_RS01805, SH_RS01810, SH_RS01815, SH_RS01820, SH_RS01825, SSP_RS00305, cap8H, cap8I, cap8J, cap8K, cap8L, cap8M, cap8O, cap8P, wecB | 62 | CC3, ST2, CC5 | type 8 capsular polysaccharide synthesis |
| GCA_044003545_10-226904-243615 | PAI_25 | GCA_044003545 | GCA_044003545_10 | 226904 | 243615 | 16,712 | SH_RS01805, SH_RS01810, SH_RS01815, SH_RS01820, SH_RS01825, SSP_RS00305, cap8H, cap8I, cap8J, cap8K, cap8L, cap8M, cap8O, cap8P, wecB | 135 | all | type 8 capsular polysaccharide synthesis |
| GCA_022689325_2-6446-13541 | PAI_26 | GCA_022689325 | GCA_022689325_2 | 6446 | 13541 | 7,096 | SH_RS01805, SH_RS01810, SH_RS01815, SSP_RS00305, cap8O, cap8P | 138 | all | type 8 capsular polysaccharide synthesis |
| GCA_008728815_1-2992-17125 | PAI_27 | GCA_008728815 | GCA_008728815_1 | 2992 | 17125 | 14,134 | SH_RS01860, aaa, aae | 144 | all | autolysin/adhesin |
| GCA_044003545_1-67165-81398 | PAI_28 | GCA_044003545 | GCA_044003545_1 | 67165 | 81398 | 14,234 | SH_RS01860, aaa, aae | 144 | all | autolysin/adhesin |
| GCA_000025085_1-1725148-1758900 | ICE_01 | GCA_000025085 | GCA_000025085_1 | 1725148 | 1758900 | 33,753 | rve (intergrase), ftsY (relaxase), trwB (T4CP) | 144 | all |  |
| GCA_000542395_1-144833-238371 | ICE_02 | GCA_000542395 | GCA_000542395_1 | 144833 | 238371 | 93,539 | nirQ (T4SS), asd (T4SS), virB4 (T4SS), virB3 (T4SS), virB11 (T4SS) | 144 | all |  |
| GCA_020682785_1-286431-346386 | ICE_03 | GCA_020682785 | GCA_020682785_1 | 286431 | 346386 | 59,956 | yxdL (T4SS), ssaA (T4SS), prgC (T4SS) | 144 | all |  |
| GCA_022568915_1-369516-412540 | ICE_04 | GCA_022568915 | GCA_022568915_1 | 369516 | 412540 | 43,025 | xerD (integrase), prgIc (T4SS), comGA (T4SS) | 144 | all |  |
| GCA_022568985_1-567091-601765 | ICE_05 | GCA_022568985 | GCA_022568985_1 | 567091 | 601765 | 34,675 | xerC (integrase), trsG (T4SS), trbC (T4SS) | 144 | all |  |
| GCA_022569055_1-726185-803995 | ICE_06 | GCA_022569055 | GCA_022569055_1 | 726185 | 803995 | 77,811 | xerC (integrase), hslU (T4CP), ftsK (T4CP), rny (T4SS), glpF (T4SS), miaA (T4SS) | 144 | all |  |
| GCA_022569245_1-367284-404262 | ICE_07 | GCA_022569245 | GCA_022569245_1 | 367284 | 404262 | 36,979 | integrase, traK (T4SS), tfc10 (T4SS), yciC (T4SS) | 63 | CC1, ST27, ST4/ST13, CC28, CC5, CC3 |  |
| GCA_022689385_1-9854-59056 | ICE_08 | GCA_022689385 | GCA_022689385_1 | 9854 | 59056 | 49,203 | prgK (T4SS), bceA (T4SS), fepC (T4SS), bmrA (T4SS), tagH (T4SS), traK (T4SS), virB4 (T4SS), trbC (T4SS), integrase | 144 | all |  |
| GCA_032480715_1-47344-120215 | ICE_09 | GCA_032480715 | GCA_032480715_1 | 47344 | 120215 | 72,872 | tfc14 (T4SS), lspA (T4SS), trwB (T4CP), rbgA (T4SS), xerC (integrase) | 144 | all |  |
| GCA_040717915_1-917693-939664 | ICE_10 | GCA_040717915 | GCA_040717915_1 | 917693 | 939664 | 21,972 | tfc24 (T4SS), yknY (T4SS), ychF (T4SS), integrase | 144 | all |  |
| GCA_020882195_1-1470466-1524939 | Prophage_01 | GCA_020882195 | GCA_020882195_1 | 1470466 | 1524939 | 54,474 | IS256, yorK, satA, aphA, aacA-aphD, dnaE, cmoA, hhaIM | 2 | CC1 |  |
| GCA_032564755_9-200-28813 | Prophage_02 | GCA_032564755 | GCA_032564755_9 | 200 | 28813 | 28,614 | nrdI, nrdE, nrdF, rnhA, yorK, aph(3')-III, aac(6')-aph(2'') | 1 | CC1 | tolerance to oxidative stress |
